# Interpositional Arthroplasty With Dermal Grafting and Hinged External Fixator for Elbow Osteoarthrosis Associated With Stiffness in Young Adults

**DOI:** 10.7759/cureus.81723

**Published:** 2025-04-04

**Authors:** Luis Alfredo Gómez-Vieira, Gisele Florence C Azevedo-Gómez, Jorge Assunção, Mauro E Gracitelli, Fernando B Andrade-Silva, Arnaldo A Ferreira Neto, Eduardo A Malavolta

**Affiliations:** 1 Orthopedics, Hospital Mater Dei, Salvador, BRA; 2 Radiology, Hospital Português da Bahia, Salvador, BRA; 3 Orthopedics and Traumatology, Hospital das Clínicas da Faculdade de Medicina da Universidade de São Paulo, São Paulo, BRA

**Keywords:** arthroplasty, contracture, elbow joint, joint capsule release, treatment outcome, young adults

## Abstract

Introduction

Elbow stiffness can be disabling when performing activities. Surgery is indicated when conservative treatment fails. In older patients, the most appropriate surgical option is total elbow arthroplasty. However, in younger patients, the choice of surgery remains a topic of discussion.

Materials and methods

This study was a retrospective case series with a five-year follow-up, evaluating the outcomes of interpositional arthroplasty with dermal grafting and a hinged external fixator for elbow osteoarthrosis associated with stiffness in young adults according to the Mayo Elbow Performance Score (MEPS), the quickDASH score, the Visual Analogue Scale (VAS), and range of motion (ROM). Outcomes were assessed preoperatively and at 3, 6, 12, 24, and 60 months postoperatively. Imaging evaluations were performed preoperatively and at the 60-month follow-up. All complications were documented.

Results

We evaluated 40 patients. The MEPS increased from 42.6 ± 12.2 to 70.3 ± 18.6 (p < 0.001). The quickDASH score decreased from 43.4 ± 3.2 to 25.3 ± 9.7 (p < 0.001), and the VAS score decreased from 8.7 ± 2.0 to 3.7 ± 2.7 at the end of follow-up (p < 0.001). The flexion-extension arc increased from 49˚ ± 27˚ to 92˚ ± 29˚ (p < 0.001). Imaging studies showed a reduction in joint narrowing, a decrease in osteophytes, a higher radiographic classification of arthrosis, and fewer loose bodies. A total of 65% of patients experienced transient complications, while 5% had complications requiring reoperation, including arthrodesis and total elbow arthroplasty.

Conclusions

Interpositional arthroplasty with dermal grafting and a hinged external fixator for elbow osteoarthrosis associated with stiffness in young adults significantly improves clinical functional scale scores, ROM, and radiological appearance at the five-year follow-up. Despite the high rate of complications, most were transient and manageable, with only a small percentage of cases (5%) requiring surgical reintervention. The procedure demonstrated sustained functional benefits over time, with early improvements in pain and mobility stabilizing in the long term.

## Introduction

Elbow stiffness can be disabling when performing basic daily activities [1,2]. Surgery is indicated when conservative treatment fails and may improve the patient’s range of motion (ROM) and quality of life [3-7].

Total elbow arthroplasty is the best surgical option for patients older than 65 [8]. In younger patients, the optimal surgical approach remains a subject of debate, as no consensus has been reached [9,10]. Interpositional arthroplasty may relieve pain and improve elbow ROM while preserving regional bone stock and facilitating future surgical reconstruction options [11]. This procedure is a viable surgical option, particularly for young patients [12]. However, few studies with large patient cohorts have assessed its medium- and long-term outcomes [4,9].

Interpositional elbow arthroplasty achieves good results in 70% of patients with elbow stiffness. This surgical technique can be performed using various tissue types, such as fascia lata [9], dermis [13], Achilles tendon allograft [13], anconeus [14], triceps [15], bovine collagen membrane [16], silicone [17], gelfoam [18], and AlloDerm [19].

This study aimed to evaluate the clinical outcomes of interpositional arthroplasty with an autologous dermal graft and an articulated external fixator for elbow stiffness over a five-year follow-up period. We hypothesize that interpositional arthroplasty with dermal grafting and a hinged external fixator for elbow osteoarthrosis associated with stiffness in young adults can improve clinical functional scales and elbow ROM at five years of follow-up.

## Materials and methods

Study design

In this retrospective study, we analyzed patients with elbow stiffness who underwent open surgical elbow release combined with interpositional arthroplasty using autologous dermal grafts and a hinged external fixator. All procedures were performed at the lead author's institution between January 2006 and April 2016. Although the study design was retrospective, clinical data were prospectively collected at standardized postoperative intervals as part of the institution’s routine follow-up protocol. The Institutional Review Board of the Faculty of Medicine of the University of São Paulo issued approval 08309419.4.0000.0065.

Participants

Young patients (aged 20 to 50 years) with elbow stiffness, defined as a flexion-extension ROM of less than 100°, a maximum extension of less than 30°, and a maximum flexion of less than 130°, that did not improve after 12 months of conservative treatment and who presented with elbow arthrosis were eligible for inclusion. Patients with a history of or active infection, neurological injury, elbow pseudarthrosis, malunion, or clinically uncompensated comorbidities were excluded. Additionally, patients who discontinued follow-up before six months postoperatively were excluded. All included patients had both osteoarthrosis and associated stiffness.

Intervention

**Figure 1:**
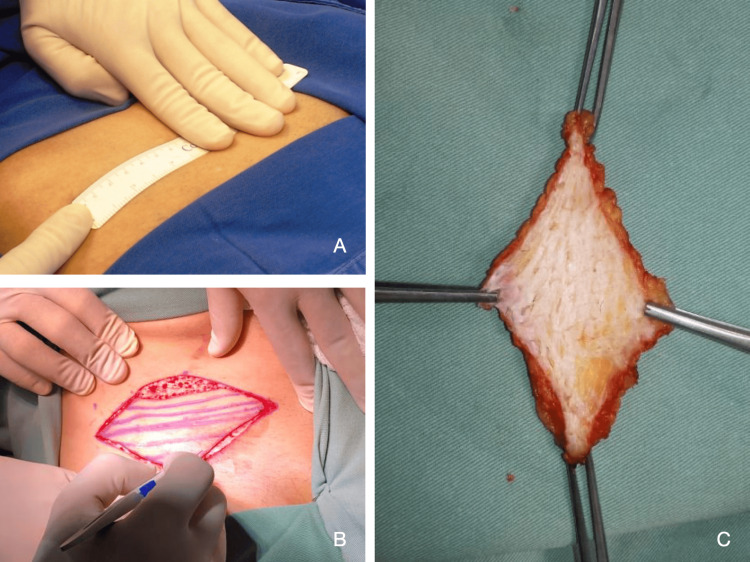
Donor area graft (A) Demarcation. (B) Preparation. (C) Autologous dermal graft prepared.

The patients underwent a brachial plexus block and general anesthesia, after which they were positioned in the supine position. The dermal graft was harvested through a transverse incision measuring 10 cm by 4 cm in the upper inguinal region (bikini line). Dissection was carried down through the subcutaneous tissue to the muscular fascia, and the dermal graft was removed en bloc. Preparation of the graft involved carefully removing the epidermis using a No. 11 scalpel blade. The donor site was then closed with an intradermal 4-0 nylon suture (Figure 1).

The dermal graft size was always the same, measuring 6 centimeters transversely and 4 centimeters longitudinally. Sterile inflatable tourniquets (250 mmHg) were applied. A global posterior incision was performed in the elbow joint, followed by lateral and medial paratricipital dissection. The ulnar nerve was identified, released, and protected. A periosteal incision was made on the medial surface of the proximal ulna, and the triceps attachment was reflected along with the forearm fascia. The extensor mechanism was reflected laterally, along with the anconeus muscle. The flexor mechanism was also reflected.

**Figure 2:**
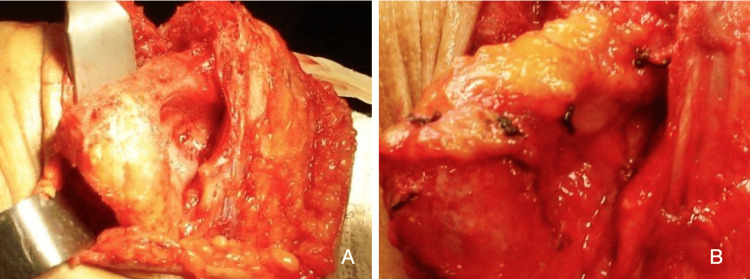
Interposition arthroplasty at the distal end of the humerus (A) Preparation and final appearance of the distal end of the humerus. (B) Final appearance of the autologous dermal graft attached to the recipient area - distal humerus end.

After releasing the anterior and posterior joint capsules, the distal end of the humerus was exposed and prepared by resecting any remaining cartilage and decorticating the region with a craper to receive the dermal autograft. All osteophytes were excised. The dermal graft was then fixed onto the distal humerus using transosseous absorbable sutures (Suturefix®). Eight 2-mm holes were drilled perpendicular to the humerus from posterior to anterior, and the graft was sutured securely in place, ensuring full contact between the graft and the prepared bone bed. No interposition was performed on the ulna (Figure 2).

**Figure 3:**
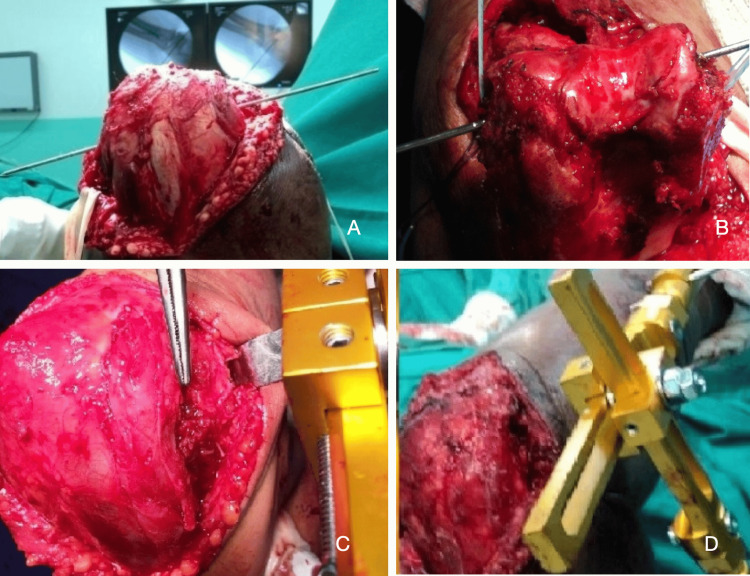
Technique for applying the dynamic external fixator to the elbow (A) Radioscopically guided guidewire placement. (B) Placement of anchors for the repair of ligaments and flexor and extensor muscles. (C) Ligaments, along with flexor and extensor tendons, were repaired. (D) A hinged external fixator was positioned at the elbow.

In most cases, we only resected the osteophytes around the radial head and preserved it. The anterior bundle of the medial collateral ligament, the lateral collateral ligament complex, and the origins of the flexor and extensor muscles were repaired or reinserted using sutures or anchors as necessary. A hinged external fixator was applied under fluoroscopic guidance to ensure joint stability and allow for early mobilization. Two 4.5 mm Schanz pins were inserted into the distal humerus, and two 4.0 mm pins were placed into the proximal ulna. A joint distraction of approximately 3 mm was applied to protect the graft and promote its integration into the decorticated distal humerus (Figure 3).

The external fixator was locked for three days after the surgery and then unlocked to assist motion and allow for pain-free motion. The ulnar nerve was transposed subcutaneously and anteriorly. The wound was closed in layers, and a 3.2 suction drain was placed to prevent hematoma. All patients were operated on by the same surgeon, the lead author of the study, who has 18 years of experience in shoulder and elbow surgery.

Postoperative treatment

Rehabilitation was initiated immediately to achieve analgesia and improve ROM according to the patient’s pain threshold. Patients were encouraged to perform common daily activities within their pain limits on the first postoperative day. The hinged external fixator was removed six weeks after surgery. Upon removal, the elbow was gently manipulated to enhance ROM. Postoperative follow-up assessments were conducted at three, six, and 12 months and two, three, four, and five years.

Outcomes and variables

The primary outcome measure was the Mayo Elbow Performance Score (MEPS) [20]. Secondary outcomes included the quickDASH score [21], the visual analog scale (VAS) score [22], ROM, radiological changes (observing the degeneration or non-degeneration evolution of the joint), and complications.

The clinical scales were applied the day before surgery, at three, six, and 12 months after surgery, and annually thereafter. Elbow ROM was recorded by manual goniometry on the day before surgery; at three, six, and 12 months after the operation; and annually thereafter.

Imaging, including radiography, computed tomography (CT), and magnetic resonance imaging (MRI), was analyzed preoperatively and at five years by a single radiologist with 18 years of experience in musculoskeletal imaging. The variables analyzed by radiography and CT included joint narrowing, osteophytosis, subluxation of the radial head, and the presence of free bodies. The Broberg-Morrey [23] and Rettig-Hastings [24] classifications were used to assess the findings from radiography. On MRI, the variables analyzed included the presence of chondropathy, synovitis, joint effusion, and ligament injury.

Complications were classified as infection, elbow instability, and ulnar nerve symptoms.

Sample size

The sample was defined by convenience and included all operated patients who met the selection criteria.

Statistical analysis

Continuous data are described by the median and the 25th and 75th percentiles, as well as by the mean and the respective standard deviation. Categorical data are presented as frequencies and proportions. The Shapiro-Wilk test was performed to verify the symmetry of the data distribution. In comparing the different evaluation times, Bonferroni-corrected p-values ≤ 0.007 were considered indicative of a statistically significant difference between the groups. Comparisons between preoperative and postoperative imaging results were performed using the McNemar test. We conducted per-protocol analyses and also performed a secondary analysis comparing patients with and without inflammatory arthropathy. Statistical analysis was carried out using SPSS Statistics version 23.0 (IBM Corp. Released 2015. IBM SPSS Statistics for Windows, Version 23.0. Armonk, NY: IBM Corp.).

## Results

Baseline characteristics

Interpositional arthroplasty with autologous dermal grafts and articulated external fixators was indicated for 48 patients. The following individuals did not meet the inclusion criteria: four patients older than 50 years, two patients younger than 20 years, one patient with active hyperthyroidism, and one patient with decompensated systemic lupus erythematosus. Therefore, 40 patients (24 men and 16 women) were included in the study. The mean age was 38.4 ± 10.1 years (Table 1).

**Table 1:** Baseline characteristics of the study population Continuous data are presented as means and standard deviations. Categorical data are presented as absolute numbers, with percentages in parentheses.

Baseline	N = 40
Age (yr)	38.4 ± 10.1
Male	24 (60)
Inflammatory arthropathy	11 (27.5)
Right elbow side	21 (52.5)
Dominant side	28 (70)
Previous trauma	29 (72.5)
Previous surgery	20 (50)

Clinical scales

**Figure 4:**
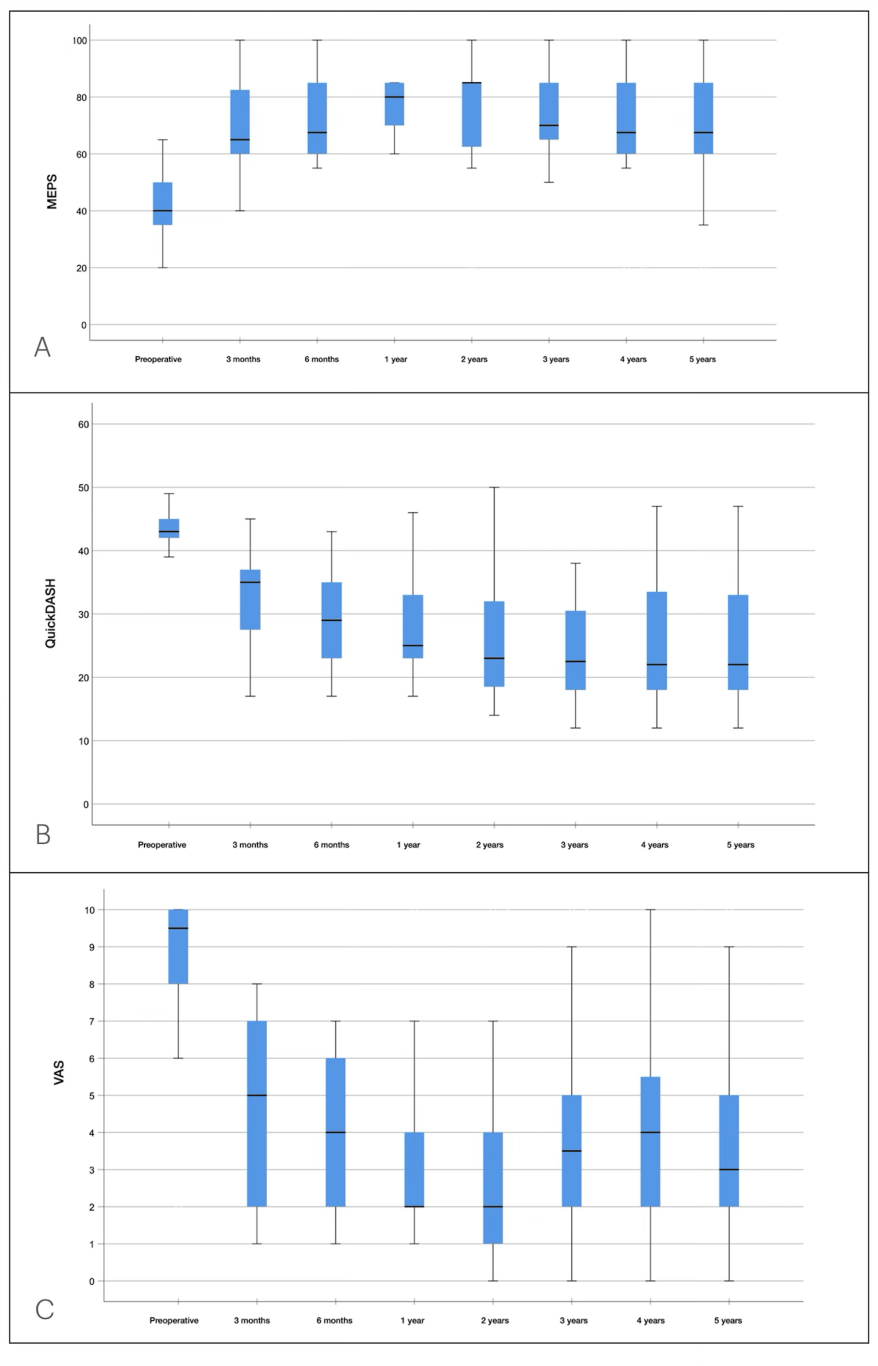
Results of clinical evaluations (A) Mayo Elbow Performance Score (MEPS). (B) QuickDASH score. (C) Visual Analogue Scale (VAS).

The mean MEPS score [20] increased from 42.6 ± 12.2 to 70.3 ± 18.6 at five years (p < 0.001). The mean quickDASH score [21] improved from 43.4 ± 3.2 to 25.3 ± 9.7 at five years (p < 0.001). The mean VAS score [22] decreased from 8.7 ± 2.0 to 3.7 ± 2.7 at the end of follow-up (p < 0.001). The MEPS [20] and quickDASH [21] scores showed statistically significant improvements up to six months postoperatively, after which no further changes were observed until the end of follow-up. The VAS score [22] also improved up to six months, but at three years, it significantly worsened, after which no further changes were observed until the end of follow-up. Clinical scores are shown in Figure 4.

Range of motion

**Figure 5:**
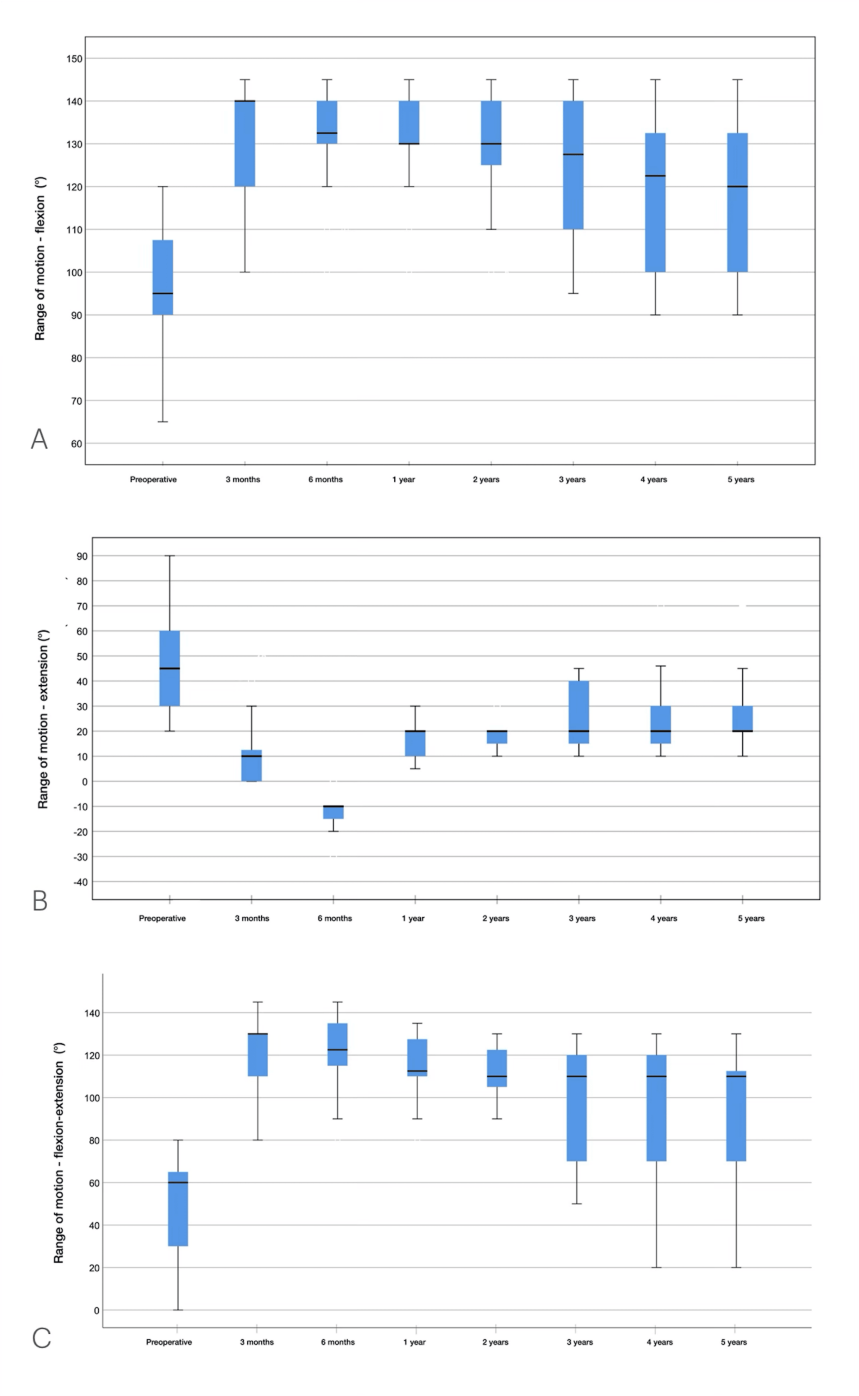
Results of flexion and extension ROM during follow-ups (A) Flexion ROM. (B) Extension ROM. (C) Flexion-extension arch. ROM: range of motion

**Figure 6:**
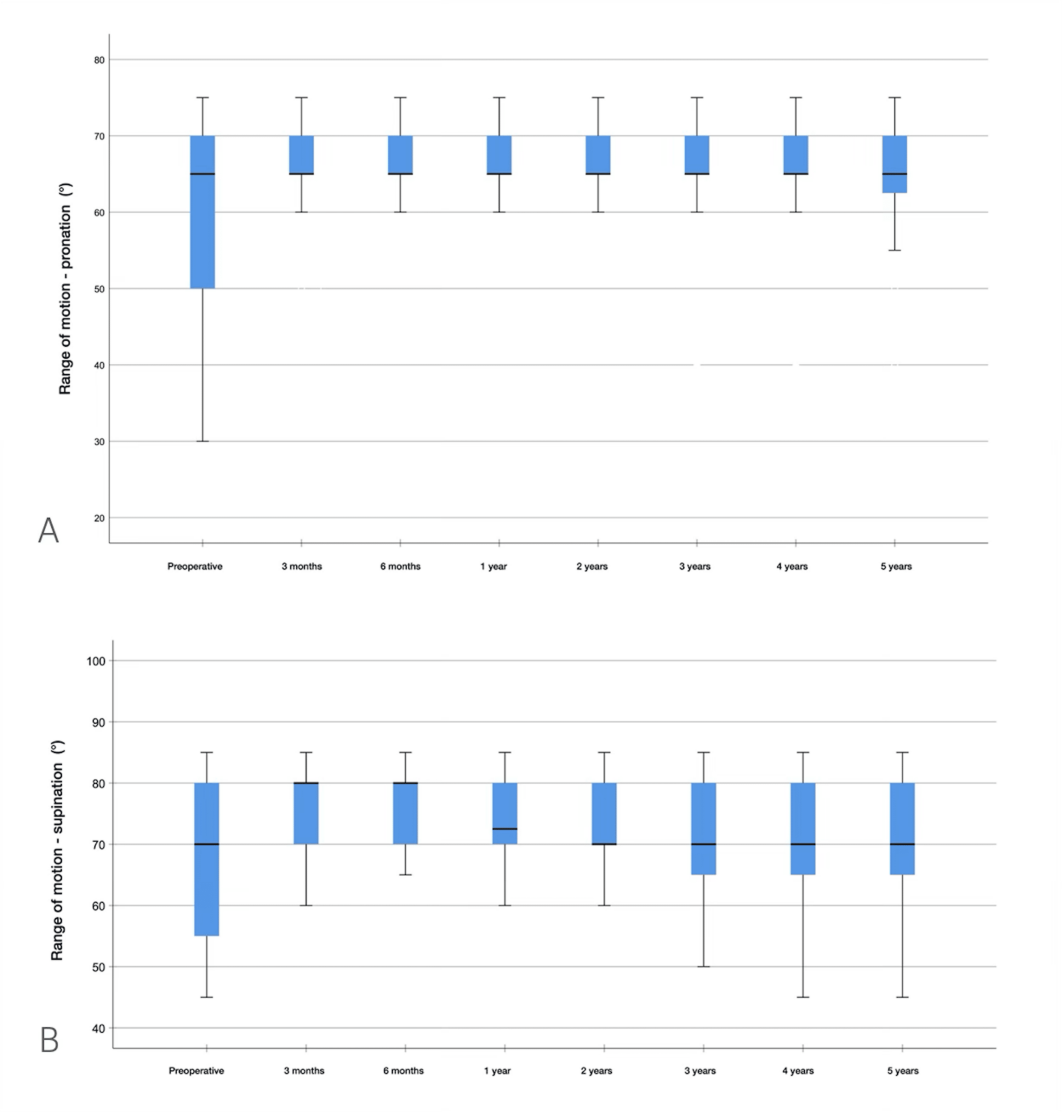
Results of pronation and supination ROM during follow-ups (A) Pronation ROM. (B) Supination ROM. ROM: range of motion

Before surgery, the mean elbow extension and flexion were 48° ± 19° and 97° ± 12°, respectively, with the mean elbow ROM being 49° ± 27°. The mean preoperative pronation arc was 60° ± 12°, the supination arc was 68° ± 13°, and the forearm rotation arc was 128° ± 24°. At the last follow-up, the mean elbow extension and flexion were 26° ± 14° and 119° ± 17°, respectively, with the mean elbow ROM being 92° ± 29° (p = 0.005). Pronation and supination did not change postoperatively. The ROM results for flexion and extension are shown in Figure 5, and pronation and supination are shown in Figure 6.

Imaging tests

X-ray

At the final follow-up, the number of patients presenting with radiocapitellar and ulnohumeral joint narrowing decreased from 27 (67.5%) to 20 (50%) and from 40 (100%) to 11 (27.5%), respectively. Our patients observed a substantial decrease in the flexion-extension ROM preoperatively. However, we did not observe the same degree of change in the pronation-supination ROM. Regarding arthrosis, there was no significant difference in its presence (p < 0.001), but its severity improved. Additionally, the rate of patients presenting with loose bodies on X-rays decreased from 30% (12 patients) to 22.5% (nine patients) (Table 2).

**Table 2:** Radiographic evaluation Categorical data are presented as absolute numbers, with percentages in parentheses.

Category		Preoperative	1 year	2 years	5 years
Radiocapitellar arthropathy		27 (67.5)	25 (62.5)	24 (60)	18 (54.5)
Ulnohumeral arthropathy		40 (100)	0 (0)	2 (5)	11 (33.3)
Osteophytosis		40 (100)	0 (0)	6 (15)	13 (39.4)
Radiocapitellar subluxation		3 (7.5)	0 (0)	0 (0)	3 (9.1)
Loose bodies		12 (30.5)	7 (17.5)	8 (20)	7 (17.5)
Arthropathy	I	12 (30.5)	33 (82.5)	32 (80)	18 (54.5)
	II	21 (52.5)	5 (12.5)	4 (10)	12 (36.4)
	III	7 (17.5)	2 (5)	4 (10)	3 (9.1)
Broberg-Morrey	I	12 (30.5)	33 (82.5)	33 (82.5)	20 (60.6)
	II	22 (55)	5 (12.5)	4 (10)	9 (27.3)
	III	6 (15)	2 (5)	3 (7.5)	4 (12.1)
Hasting-Rettig	I	12 (30)	32 (80)	32 (80)	19 (57.6)
	II	25 (62.5)	8 (20)	7 (17.5)	13 (39.4)
	III	3 (7.5)	0 (0)	1 (2.5)	1 (2.5)

Computed Tomography

At the final follow-up, the rate of patients presenting with radiocapitellar and ulnohumeral joint narrowing on CT scans decreased from 67.5% (27 patients) to 50% (20 patients) and from 100% (40 patients) to 25% (10 patients), respectively. Arthrosis showed no significant difference in incidence (p < 0.001) but improved in severity, and the incidence of loose bodies decreased from 12 (30%) to nine patients (22.5%) (Table 3).

**Table 3:** CT and MRI evaluation Categorical data are presented as absolute numbers, with percentages in parentheses. CT: computed tomography, MRI: magnetic resonance imaging

	Preoperative	1 year	2 years	5 years
CT scan				
Radiocapitellar arthropathy	27 (62.5)	24 (60)	25 (62.5)	17 (53.1)
Ulnohumeral arthropathy	40 (100)	0 (0)	2 (5)	9 (28.1)
Osteophytosis	40 (100)	0 (0)	6 (15)	11 (27.5)
Radiocapitellar subluxation	3 (7.5)	0 (0)	0 (0)	3 (9.4)
Loose bodies	12 (30)	7 (17.5)	8 (20)	7 (17.5)
Arthropathy I	12 (30)	33 (82.5)	32 (80)	17 (53.1)
Arthropathy II	21 (52.5)	5 (12.5)	4 (10)	11 (27.5)
Arthropathy III	7 (17.5)	2 (5)	4 (10)	2 (5)
MRI				
Chondropathy	40 (100)	40 (100)	40 (100)	32 (80)
Synovitis	14 (35)	7 (17.5)	8 (20)	9 (28.1)
Articular effusion	15 (37.5)	2 (5)	4 (10)	8 (20)
Ligament tear	1 (2.5)	2 (5)	3 (7.5)	4 (9.4)
Tendon tear	0 (0)	0 (0)	0 (0)	0 (0)

Magnetic Resonance Imaging

At the final follow-up, chondropathy of the elbow persisted in 100% (40 patients), the incidence of synovitis decreased from 30% (12 patients) to 27.5% (11 patients), the incidence of joint effusion decreased from 37.5% (15 patients) to 22.5% (nine patients), and the incidence of ligament injury increased from 2.5% (one patient) to 10% (four patients) (Table 3).

Complications

A total of 26 patients (65%) experienced transient complications, and two patients (5%) had complications that compromised the final result of the surgical procedure. One patient underwent arthrodesis, and the other underwent total elbow arthroplasty. All 10 patients (25%) with transient ulnar nerve neuropathy also exhibited residual joint instability. Additionally, three patients (7.5%) developed superficial infections at the site of the pins of the articulated external fixator. These infections were treated with daily local asepsis and oral antibiotics, resulting in resolution. Two patients (5%) required reoperations. One patient (2.5%), due to residual joint instability, underwent ligament reconstruction with the palmar tendon while still exhibiting gross elbow instability and was referred for total elbow arthroplasty. The second patient presented with a late deep infection and underwent surgical cleaning without satisfactory improvement, eventually progressing to arthrodesis (Table 4).

**Table 4:** Complications and reinterventions n: absolute numbers of complications or patients

Complications	n	%
Ulnar nerve neuropraxia	10	25
Elbow instability	11	27.5
Deep infection	1	2.5
Superficial infection	7	17.5
Patients with complications	18	45
Need for surgical reintervention	2	5

Secondary analysis

Per-Protocol Analysis

This secondary analysis included 38 patients who completed the five-year follow-up without requiring arthrodesis or total elbow arthroplasty. They demonstrated an improvement in the MEPS score [20], with a mean of 42.6 ± 12.1 preoperatively and 70.3 ± 18.6 at five years (p < 0.001). The improvement occurred within the first six months of follow-up. Regarding ROM, the flexion and extension arcs increased from a mean of 97° ± 12° and 47° ± 19° preoperatively to a mean of 120° ± 17° and 25° ± 11°, respectively, at five years (p < 0.001).

Inflammatory Arthropathy

Both patients without inflammatory arthropathy (29 patients) and those with the condition (11 patients) showed improvement after the procedure. The mean MEPS score [20] changed from 44.5 ± 12.2 and 37.7 ± 10.8 preoperatively, respectively, to 74.5 ± 15.0 and 59.1 ± 23.0 at five years; the difference over time was statistically significant (p < 0.001), but there was no statistically significant difference between the groups. The mean preoperative flexion-extension ROM was 52° ± 27° and 42° ± 25°, respectively, increasing to 104° ± 20° and 61° ± 26° at five years of follow-up, with a statistically significant change over time (p < 0.001).

## Discussion

Our results showed that interpositional arthroplasty with a dermal graft, combined with a hinged external fixator, in the treatment of stiff elbow arthropathy in young adults may lead to improvements in functional scores and ROM at five years of follow-up. Among the 40 patients included in the study, only two required surgical revision: one underwent elbow arthrodesis due to a late deep infection, and the other was converted to total elbow arthroplasty following persistent instability despite ligament reconstruction. In our clinical practice, isolated surgical release without interposition has yielded unsatisfactory outcomes in patients with severe joint degeneration. Although previous studies have shown good results with isolated release in cases of elbow stiffness without significant arthropathy, these represent distinct clinical conditions. For young patients with advanced post-traumatic arthropathy, arthrodesis or total elbow arthroplasty are often the only alternatives, both of which have considerable limitations. The combined technique we adopted offers a joint-preserving option that aims to restore function, relieve pain, and potentially delay or avoid more invasive salvage procedures.

Regarding the MEPS [20], there was a statistically significant improvement over the first six months, after which it remained relatively unchanged. Notably, the patient samples are heterogeneous, making comparisons between the various studies difficult. Nevertheless, our results are superior to those achieved by some authors [10,25-26] and lower than those achieved by others [27,28]. The same pattern applies to the quickDASH score [21], with improvement in the first semester followed by stability afterward. Lindenhovius et al. [29] observed an improvement in the DASH score [18] that was greater than our results after performing open surgical release of the elbow. However, the follow-up was shorter, and it is not possible to confirm that the results would be identical in the medium and long term. Additionally, no interposition arthroplasty was performed, which suggests that the elbows of the patients had better articular cartilage. Erşen et al. [30] performed interposition with the Achilles tendon and observed an improvement in the DASH score [18] that was greater than our results. However, their sample was smaller than ours, and it is impossible to state with certainty that their results would remain the same if applied to more patients. The improvement in the VAS score [22] over 24 months was statistically significant in our sample. However, at 36 months, it worsened slightly and remained stable from then on. Our result is similar to that of Laubscher et al. [31] and better than Sun et al. [32]. Gracitelli et al. [25], in turn, did not notice a statistically significant improvement in this scale, although they performed stiffness release without interposition.

The elbow flexion-extension ROM improved significantly, reaching a maximum between three and six months, with slight worsening thereafter. This reinforces the importance of medium- and long-term evaluations. Other authors also observed a progressive worsening of this outcome during follow-up [27]. The values we observed until the sixth month of treatment were slightly higher than those reported in the systematic review by Kodde et al. [33].

Few studies [24,34,35] have evaluated the radiological parameters of stiff elbows. In this study, X-ray and CT evaluations showed changes in various parameters that did not follow the standard for joint degeneration from the preoperative period to the end of follow-up.

Rettig et al. [24], with a minimum follow-up of two years, observed that 12% of 17 operated elbows showed worsening of joint degeneration according to their classification, transitioning from Class I to Class II or from Class II to Class III. In our study, we observed an increase in Class I patients (62.5%) and a decrease in Class II patients (35%). We also observed an improvement in the arthrosis patterns of our patients, with a decrease in severe cases (17.5% to 7.5%) and an increase in mild cases (30% to 60%). Guitton et al. [34] presented only one postoperative analysis, which found that 23% of the patients had radiographic evidence of moderate or severe arthrosis of the elbow. In contrast, 54% had mild arthrosis at the end of the follow-up. Analyzing osteophytosis on X-ray and CT, our study observed a recurrence rate of 32.5%, whereas Rettig et al. [24] identified a recurrence rate of 54%. Regarding radiocapitellar narrowing, unlike Rettig et al. [24], who showed that 100% of their patients worsened, we observed improvement in 17.5% of our patients.

The rate of Class I is high (30%). There is also a high rate of patients with minimal osteoarthrosis. What we expected to find is not always what we actually find. At the beginning of surgical planning for these patients, we did not anticipate discovering chondral compromise that would justify interposition arthroplasty. However, that is exactly what we found, and the interpositions were necessary.

According to Lombard et al. [35], CT is superior to radiography in identifying and characterizing the bony causes of elbow stiffness. In contrast, MRI has a limited role in evaluating elbow stiffness and may not be necessary.

The parameters evaluated on MRI showed persistent and consistent changes from the preoperative period to the five-year follow-up. We did not find studies that analyzed the results of this imaging modality in young adult patients with elbow stiffness.

Like Hausman and Birnbaum [19], the external fixator was applied by introducing distraction to the device to protect the graft and facilitate its bone integration. This approach was also supported by Akhtar et al. [4], and in Nolla et al. [36], a joint distraction of 3-4 millimeters was established, allowing for a stable elbow and enabling rehabilitation to begin as soon as possible.

One of the most common complications in interpositional elbow arthroplasty is ulnar nerve neuropraxia. Our study presented findings similar to those of Antuña et al. [37], with 25% of patients reporting symptoms related to the ulnar nerve. Cohen and Hastings [5] and Gelinas et al. [38] described a lower incidence, 14% and 9%, respectively. As in most studies, our patients eventually presented with complete regression of symptoms.

We had one patient with a deep infection (2.5%), which resulted in the only case requiring arthrodesis by the end of treatment. Tan et al. [39] reported three cases (5.8%) that were resolved after successive surgical cleanings. Regarding superficial infection, unlike Gracitelli et al. [25], who reported one case of surgical wound infection (3.8%), this condition appeared in seven of our patients (17.5%), all in the path of the articulated external fixator pins. Antibiotic therapy resolved these infections similarly to the method described by Laubscher et al. [31].

Similar to other authors [36,40], we applied distraction to the external fixator to protect the graft. The assembly allowed for the early initiation of rehabilitation, providing a stable elbow.

In the study by Laubscher et al. [31], with a mean follow-up of 4.5 years, 11.7% of the patients with residual elbow instability improved without the need for surgical reintervention. In our study, 11 patients (27.5%) presented this complication; in the majority (22.5%), it did not compromise the course of the patient's recovery.

Our study has some limitations. The lack of a control group and the relatively small sample size limit the analysis of secondary outcomes and reduce the generalizability of the findings. Additionally, although mostly transient, the high rate of complications may reflect the pathology's complexity and the procedure's technical demands. These factors should be considered when interpreting the results, as they may influence the applicability of the technique in broader clinical settings.

As strengths, standardized clinical scales were applied, allowing for an adequate evaluation of the clinical and radiological evolution of the patients. Another advantage was that the patients were evaluated sequentially until the fifth year, enabling a consistent assessment of the data in the medium term. Although studies have shown no statistically significant difference after 6 months of treatment [4,41], medium- and long-term follow-up are crucial for understanding the lesions' evolution and anticipating the disease's progression and its treatment.

Given the complexity of treating post-traumatic elbow arthropathy in young patients, our findings support the use of interpositional arthroplasty with a dermal graft and hinged external fixation as a viable alternative for those who are not candidates for total elbow arthroplasty. The clinical applicability of this approach lies in its ability to relieve pain, improve the ROM, and preserve joint integrity while postponing more definitive salvage procedures. Future research should focus on refining the surgical technique, particularly in minimizing complications such as residual instability and transient neuropathies. Additionally, prospective controlled studies with larger samples and longer follow-up are needed to better define patient selection criteria and optimize postoperative rehabilitation protocols aimed at reducing complication rates and enhancing long-term outcomes.

## Conclusions

Interpositional arthroplasty with dermal grafting and a hinged external fixator for elbow osteoarthrosis associated with stiffness in young adults leads to significant improvements in clinical functional scale scores, ROM, and radiological appearance at the five-year follow-up. Despite the high rate of complications, most were transient and manageable, with only a small number of cases (5%) requiring surgical reintervention. The procedure demonstrated sustained functional benefits over time, with early improvements in pain and mobility stabilizing in the long term.
